# Pufferfish gasdermin Ea is a significant player in the defense against bacterial pathogens

**DOI:** 10.1007/s42995-024-00237-x

**Published:** 2024-06-28

**Authors:** Hang Xu, Kunpeng Qin, Kangwei Hao, Zihao Yuan, Li Sun

**Affiliations:** 1 CAS and Shandong Province Key Laboratory of Experimental Marine Biology, Institute of Oceanology, CAS Center for Ocean Mega-Science, Chinese Academy of Sciences, Qingdao, 266071 China; 2 Laboratory for Marine Biology and Biotechnology, Laoshan Laboratory, Qingdao, 266237 China; 3 School of Marine Science, University of Chinese Academy of Sciences, Qingdao, 266400 China

**Keywords:** GSDME, Caspase, *Takifugu rubripes*, Bacterial infection, Immune defense

## Abstract

Gasdermins (GSDMs) are proteins cleaved by caspase (CASP) to trigger pyroptosis. In teleosts, pyroptosis is mediated by gasdermin E (GSDME). The Pufferfish, *Takifugu rubripes*, possesses two GSDME orthologs: named TrGSDMEa and TrGSDMEb. TrGSDMEa is cleaved by CASP3/7 to liberate the N-terminal (NT) domain that can trigger pyroptosis in mammalian cells. However, the biological function of TrGSDMEa in pufferfish is unknown, and TrGSDMEb is poorly studied. We found that TrGSDMEb was cleaved by CASP1/3/6/7/8, but the resulting NT domain, despite its similarity to TrGSDMEa-NT domain in sequence and structure, failed to induce pyroptosis. TrGSDMEa and TrGSDMEb exhibited similar expression patterns in pufferfish under normal physiological conditions but were up- and downregulated, respectively, in expression during *Vibrio harveyi* and *Edwardsiella tarda* infection. Bacterial infection induced the activation of TrGSDMEa and CASP3/7 in pufferfish cells, resulting in pyroptosis accompanied with IL-1β production and maturation. Inhibition of TrGSDMEa-mediated pyroptosis via TrCASP3/7 reduced the death of pufferfish cells and augmented bacterial dissemination in fish tissues. Structure-oriented mutagenesis identified 16 conserved residues in teleost GSDMEa that were required for the pore formation or auto-inhibition of GSDMEa. This study illustrates the role of GSDMEa-mediated pyroptosis in teleost defense against bacterial pathogens and provides new insights into the structure-based function of vertebrate GSDME.

**Supplementary Information:**

The online version contains supplementary material available at 10.1007/s42995-024-00237-x.

## Introduction

Gasdermins (GSDMs) are pore-forming proteins that can directly trigger pyroptosis, a type of inflammatory lytic cell death (Broz et al. 2020; De Schutter et al. 2021). In humans, the GSDM family has six members: GSDMA, GSDMB, GSDMC, GSDMD, GSDME, and PJVK (also known as DFNB59) (Shi et al. 2017; Tamura et al. 2007). Except for PJVK, all GSDMs have an N-terminal (NT) cytotoxic domain and a C-terminal (CT) inhibitory domain connected by a linker region (De Schutter et al. 2021; Shi et al. 2017). During pyroptosis, GSDM is cleaved by a protease at the linker region to release the NT domain from the auto-inhibitory CT domain. The lipophilic NT domain targets the plasma membrane via interaction with membrane lipids and eventually forms pores that lead to cell rupture (Ding et al. 2016; Kesavardhana et al. 2020; Kovacs and Miao 2017).

In mammals, GSDMD can be cleaved by inflammatory caspase (CASP) via two mechanisms. CASP1 and CASP4/5 in humans, or CASP1 and CASP11 in mice, are activated by canonical inflammasome and bacterial lipopolysaccharide (LPS), respectively (Chen et al. 2016; He et al. 2015; Kayagaki et al. 2015; Shi et al. 2015). These CASPs cleave GSDMD at a tetrapeptide (FLTD in humans and LLSD in mice) in the linker region. This produces a pore-forming NT fragment that forms pores in the cytoplasmic membrane and results in the release of inflammatory cytokines, such as interleukin (IL)-1β and IL-18, to amplify the inflammation (Chen et al. 2016; He et al. 2015; Kayagaki et al. 2015; Shi et al. 2015; Xia et al. 2021). As opposed to GSDMD, which is present only in mammals, GSDME can be traced back to invertebrates, such as cnidaria and molluscs (Chen et al. 2023; Jiang et al. 2020; Qin et al. 2023). In mammals, GSDME is cleaved by CASP3 at the linker region to induce pyroptosis (Rogers et al. 2017; Wang et al. 2017). In contrast, various cleavage patterns of GSDME exist in teleost (Hofmann 2020; Yuan et al. 2022).

Fish GSDME has undergone genetic diversification and exhibits three orthologs: GSDMEa, GSDMEb, and GSDMEc (Yuan et al. 2022). The GSDMEa of the zebrafish *Danio rerio* and turbot *Scophthalmus maximus* are activated by CASP3/7 cleavage (Wang et al. 2017; Xu et al. 2022a), while the GSDMEb of the zebrafish and tongue sole *Cynoglossus semilaevis* are activated by caspy2 (CASP4/5 homolog) and CASP1, respectively (Jiang et al. 2019; Wang et al. 2020). GSDM is an important molecule in anti-infection immune defense (Liu et al. 2021; Magnani et al. 2022). For example, GSDMD deficiency increases the susceptibility of mice to bacterial infections by *Francisella novicida* and *Burkholderia pseudomallei* (Wang et al. 2019; Zhu et al. 2018). The functional study of teleost GSDME is limited to turbot and common carp *Cyprinus carpio*, with turbot GSDMEa and common carp GSDMEb being involved in the response to bacterial infection (Xu et al. 2022a; Zhao et al. 2023). Here, we extend the study of GSDME by examining the role of GSDMEa in bacterial infection using pufferfish as a model.

The Pufferfish, *Takifugu rubripes*, is an economically important teleost species in Asia, especially in Japan and China. Pufferfish possess the smallest vertebrate genome (~ 400 Mb), but they harbor genes and regulatory sequences similar to those found in other vertebrates (Aparicio et al. 2002; Yamanoue et al. 2009). Therefore, pufferfish can be used as a model organism for studying genome architecture and function (Aparicio et al. 2002; Brenner et al. 1993; Huang et al. 2021; Yamanoue et al. 2009). GSDMEa and GSDMEb are present in the pufferfish genome, but their activity and function are unknown. In the present study, we examined the cleavage mechanism and pyroptosis-inducing capacity of pufferfish GSDMEb and determined the role of GSDMEa in the immune defense against the fish bacterial pathogens *Vibrio harveyi* and *Edwardsiella tarda*. We also reveal the molecular mechanism of the pore-forming and auto-inhibition capacities of GSDMEa in this teleost. These findings aid our understanding of the immune function and working mechanism of teleost GSDME.

## Materials and methods

### Animal, cell line, and bacteria

Pufferfish were obtained from a fish farm in Rizhao (Shandong, China) and maintained at 15 °C in aerated seawater (35 PSU) for 14 days as reported previously (Zhang et al. 2023). *V. harveyi* and *E. tarda* were cultured at 28 °C with shaking as described previously (Zhang et al. 2008a, b). HEK293T cells were obtained from the ATCC (Rockville, MD, USA) and grown in DMEM medium (Corning, NY, USA) supplemented with 10% FBS (Gibco, Renfrewshire, UK) at 37 °C and 5% CO_2_.

### Sequence analysis

GSDME sequences were collected from the NCBI Reference Sequence Database (http://www.ncbi.nlm.nih.gov/RefSeq/). Phylogenetic analysis was performed as reported previously (Xu et al. 2022a) and edited with iTOL. Sequence alignment was performed using the Clustal W program, and images were generated with ESPript 3.0 (http://espript.ibcp.fr/ESPript/cgi-bin/ESPript.cgi). Three-dimensional (3D) models were produced with the Robetta server (http://robetta.bakerlab.org/) and visualized by the VDM program (http://www.ks.uiuc.edu/Research/vmd/).

### Gene cloning and mutation

Tissues were collected from pufferfish under aseptic conditions and used for total RNA isolation with FastPure Cell/Tissue Total RNA Isolation Kit V2 (Vazyme Biotech Co., Ltd., Nanjing, China). Total RNA was used for cDNA synthesis following the cDNA Synthesis Kit instruction (Thermo Fisher Scientific, Waltham, MA, USA). The coding sequences of TrGSDME and TrCASP in pufferfish were amplified by PCR. Point mutations were performed with a Hieff Mut Site-Directed Mutagenesis Kit (Yeasen, Shanghai, China) according to the manufacturer’s instructions. The primers used are listed in Supplementary Table S1.

### Quantitative real-time RT-PCR (qRT-PCR)

The qRT-PCR was conducted as previously reported (Xu et al. 2022a). Tissues were separated from pufferfish under aseptic conditions, and total RNA and cDNA were obtained as described above. The qRT-PCR was performed using ChamQ Universal SYBR qPCR Master Mix (Vazyme Biotech Co. Ltd., Nanjing, China). The relative mRNA levels were normalized by β-actin. The primers used are listed in Supplementary Table S1.

For qRT-PCR analysis during bacterial infection, 30 pufferfish were divided randomly into two groups (15 fish/tank). The infection group and the control group were intramuscularly injected with 1 × 107 CFU *V. harveyi* or an equal volume of PBS (control). *E. tarda* infection was similarly performed, except that the injection dose of bacteria was 5 × 106 CFU. Kidneys of three pufferfish from each group were collected at 6-, 12-, 24- and 48-hpi. The expressions of TrGSDMEa/b, TrCASP3/7, and cytokines were quantified by qRT-PCR as above.

### Cellular transfection

The truncates of TrGSDMEa/b were obtained as described previously (Xu et al. 2022a) and inserted into the pmCherry-N1 expression vector. For transient transfection, HEK293T cells were grown in 96-well plates (Corning, NY, USA) or 35-mm glass-bottom culture dishes (NEST Biotechnology, Wuxi, China) for 12 h. The cells were transfected with the indicated plasmid (100 ng/well or 1 μg/dish) with Lipofectamine 3000 (Invitrogen, Waltham, MA, USA). The primers used are shown in Supplementary Table S1.

### Protein purification

Recombinant human CASP1, 2, 3, 6, 7, 8, and 9 were obtained from Enzo Life Sciences (Villeurbanne, France). Recombinant TrGSDMEb, TrIL-1β, and TrCASPs were purified as described previously (Xu et al. 2022a). The coding sequences of TrGSDMEb, TrIL-1β, and TrCASPs were each cloned into a pET30a (+) vector at the BamHI and HindIII sites. The recombinant plasmids were each transformed into *Escherichia coli* Transetta (DE3). The *Escherichia coli* Transetta (DE3) cells were grown to OD_600_ 0.7 in LB medium at 37 °C with shaking, and then isopropyl-*b*-d-thiogalactopyranoside (0.2 mmol/L) was added to the culture. The culture was maintained at 15 °C for 15 h. Recombinant proteins were purified using Ni–NTA columns (GE Healthcare, Uppsala, Sweden) and dialyzed with PBS at 4 °C.

### Antibodies and immunoblotting

Polyclonal mouse anti-TrGSDMEa/b and anti-TrIL-1β antibodies were obtained as reported previously (Xu et al. 2022a). Antibodies against β-actin (AC026) were obtained from Abclonal (Wuhan, China). Immunoblotting was conducted as previously described (Li et al. 2022; Qin et al. 2023). The proteins were separated using 12% SDS–polyacrylamide gels (GenScript, Piscataway, NJ, USA) and transferred onto PVDF membranes (Millipore, Burlington, MA, USA). After blocking with 5% skim milk for 50 min, the membranes were blotted with appropriate antibodies and then with secondary antibody. The immune reactive bands were visualized with an ECL kit (Sparkjade Biotechnology Co., Ltd., Shandong, China).

### CASP activity assay

The proteolytic activity of recombinant TrCASPs was determined as reported previously (Xu et al. 2022a). Pufferfish kidney-derived macrophages and peripheral blood leukocytes were prepared and cultured in L15 medium (Sigma-Aldrich, Madrid, Spain), as described previously (Xu et al. 2022a), to determine the TrCASP3/7 activity in pufferfish cells. The cells were infected with *E. tarda* or *V. harveyi* for 4 h and 8 h in different plates and then lysed using Lysis Buffer (Solarbio, Beijing, China). The samples were treated with Ac-DEVD-AFC (MedChem Express, Monmouth Junction, NJ, USA) as reported previously (Xu et al. 2022a). The release of fluorescence was measured with BioTek Synergy HT plate reader (BioTek Instruments, Winooski, VT, USA).

### GSDME cleavage by CASP

CASP cleavage assay was conducted as previously described (Xu et al. 2022a). C-terminal His-tagged TrGSDMEb was incubated with recombinant CASPs at 24 °C in a 40-μL reaction system containing 50 mmol/L HEPES (pH 8.0), 10 mmol/L DTT, 0.005% (v/v) Tween 20, 150 mmol/L NaCl, and 3 mmol/L EDTA. The samples were analyzed with SDS-PAGE or immunoblotting with indicated antibodies after incubation for 2 h.

### Cell death assay

The cell death assay was conducted as previously described (Xu et al. 2022a). HEK293T cells were plated into 35-mm glass-bottom culture dishes for 12 h and transfected with the indicated plasmids. The cells were stained with Sytox Green (Thermo Fisher Scientific, South Logan, UT, USA) and observed with a confocal microscope (Carl Zeiss, Jena, Germany). The cells were infected with *E. tarda* for 4 h and then stained with Sytox green and CM-Dil (Yeasen, Shanghai, China) to observe pyroptosis of pufferfish macrophages. LDH release from the cells was determined using the CytoTox 96 Non-Radioactive Cytotoxicity Assay (Promega, Leiden, The Netherlands).

### TrGSDME and TrIL-1β production and cleavage in response to bacterial infection

Pufferfish kidney-derived macrophages and peripheral blood leukocytes were infected with *V. harveyi* (MOI = 1:1) or *E. tarda* (MOI = 1:1) for 4 h and 8 h in different plates. The cells and culture supernatant were treated with TCA (15% final concentration). The proteins were harvested by centrifugation and immunoblotted with indicated antibodies. β-actin was used as a loading control.

### Bacterial dissemination in fish tissues

The bacterial dissemination assay was conducted as described previously (Xu et al. 2022a, b). Pufferfish were randomly divided into two tanks (15 pufferfish/tank) and intraperitoneally (i.p.) injected with 20 μg Ac-DAVD-CHO (Science Peptide Biological Technology Co., Ltd, Shanghai, China) or an equal volume of PBS (control) for 8 h. Then, both pufferfish were infected with 1 × 10^7^ CFU *V. harveyi*. *E. tarda* infection was similarly performed, except that the injection dose of bacteria was 5 × 10^6^ CFU. At 12 and 36 hpi, spleens and kidneys were removed from the pufferfish under aseptic conditions and homogenized in sterile PBS. The homogenates were serially diluted in PBS and plated on LB plates. The bacterial recoveries on the plates were counted after incubation at 28 °C for 15 h.

### Statistical analysis

All statistical analysis was performed using GraphPad Prism 7 software. Data were analyzed with Student’s *t* test and one-way analysis of variance (ANOVA) (only for the analysis of LDH release). Statistical significance was defined as *P* < 0.05.

## Results

### Pufferfish GSDMEb is specifically cleaved by CASP1, 3, 6, 7, and 8

**Fig. 1:**
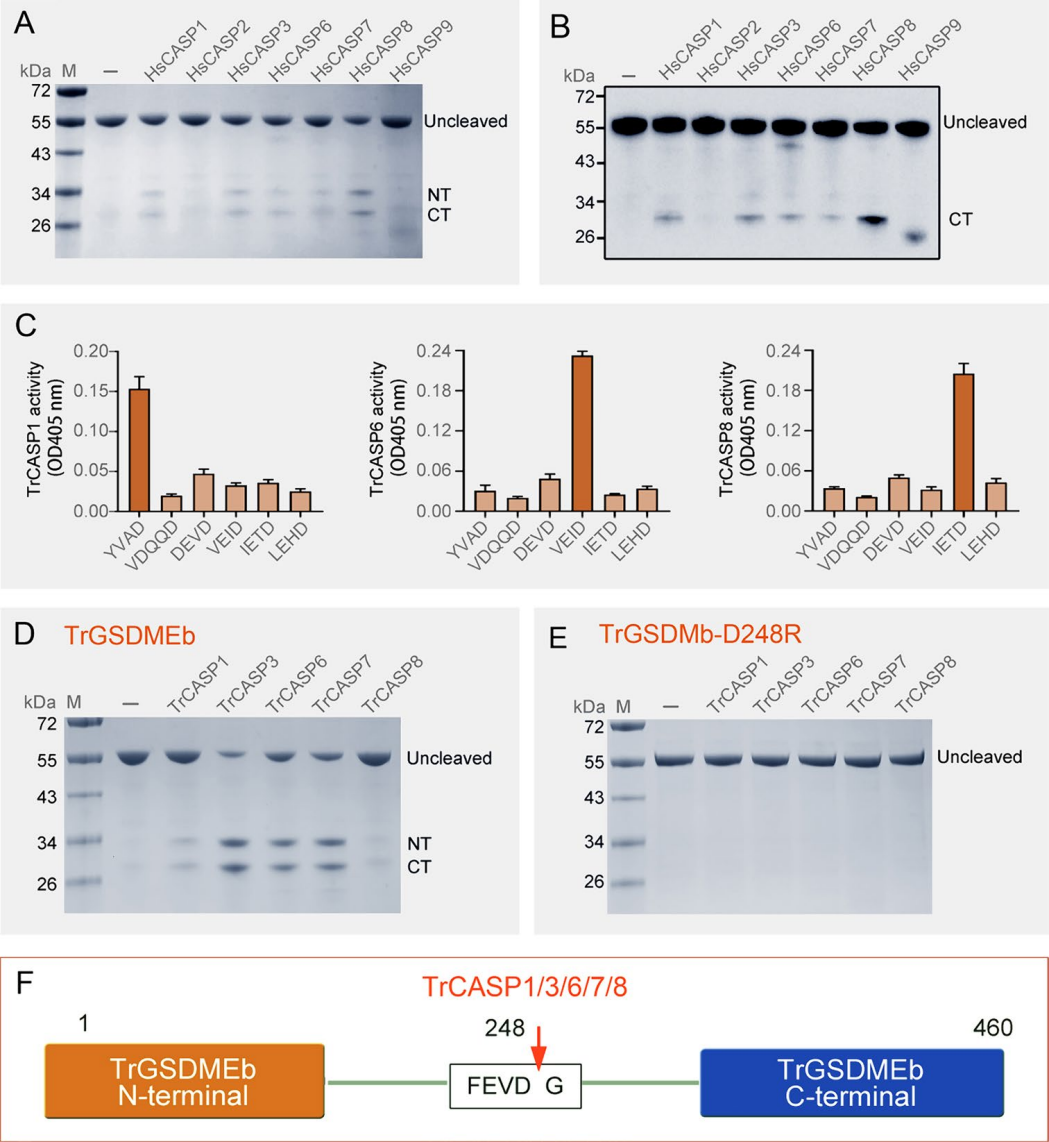
Cleavage of TrGSDMEb by caspase. TrGSDMEb was incubated with HsCASP1, 2, 3, 6, 7, 8, and 9 for 1 h and then subjected to SDS-PAGE (**A**) and immunoblotting with anti-His tag antibody (**B**). **C** The proteolytic specificities of TrCASP1, 6, and 8 were determined by treatment with different colorimetric substrates and measuring the released ρNA. The values are the means ± SD of triplicate experiments. TrGSDME (**D**) and TrGSDME-D248R (**E**) were treated with TrCASPs, and the cleavage was analyzed with SDS-PAGE. **F** Schematic diagram of TrGSDMEb cleavage by TrCASPs. The arrow indicates cleavage site. For all panels, FL, full length; NT, N-terminal fragment; CT, C-terminal fragment

Genome analysis indicated that pufferfish *Takifugu rubripes* possessed two GSDME orthologs, which were named TrGSDMEa and TrGSDMEb (Supplementary Fig. S1). We previously demonstrated that TrGSDMEa was specifically cleaved by pufferfish CASP (TrCASP) 3/7 to induce pyroptosis (Xu et al. 2024). In the present study, we found that TrGSDMEb was cleaved by recombinant human CASP (HsCASP) 1, 3, 6, 7, and 8 in similar patterns (Fig. 1A, B). To examine the cleavage potential of TrGSDMEb by pufferfish CASPs, the active forms of TrCASP1, 6, and 8 were purified (Supplementary Fig. S2). TrCASP1, 6, and 8 exhibited high proteolytic specificities toward the tetrapeptides YVAD, VEID, and IETD, respectively, which are the conserved recognition motifs of HsCASP1, 6, and 8, respectively (Fig. 1C). The cleavage specificity of TrCASP3/7 had been reported previously (Xu et al. 2024). Similar to their human counterparts, TrCASP1, 3, 6, 7, and 8 cleaved TrGSDMEb into the NT and CT fragments (Fig. 1D). A tetrapeptide motif, _245_FEVD_248_, in the vicinity of the linker region of TrGSDMEb was identified as a possible recognition site of CASP. To examine whether this motif was essential to TrCASP1, 3, 6, 7, and 8 cleavage, the mutant TrGSDMEb with D248R substitution (TrGSDMEb-D248R) was constructed. Subsequent analysis showed that TrGSDMEb-D248R was resistant to TrCASP1, 3, 6, 7, and 8 cleavage (Fig. 1E). These results indicated that TrGSDMEb was cleaved by TrCASP1, 3, 6, 7, and 8 at the _245_FEVD_248_ motif (Fig. 1F). The cleaved NT fragment of TrGSDMEb shared 41.4% identity with the TrGSDMEa-NT domain (Supplementary Fig. S3). The 3D structure of TrGSDMEb-NT was generally similar to that of TrGSDMEa-NT, but differed from the latter in certain details, such as the lack of some small α-helices (Supplementary Fig. S4).

### TrGSDMEb is unable to induce pyroptosis

**Fig. 2:**
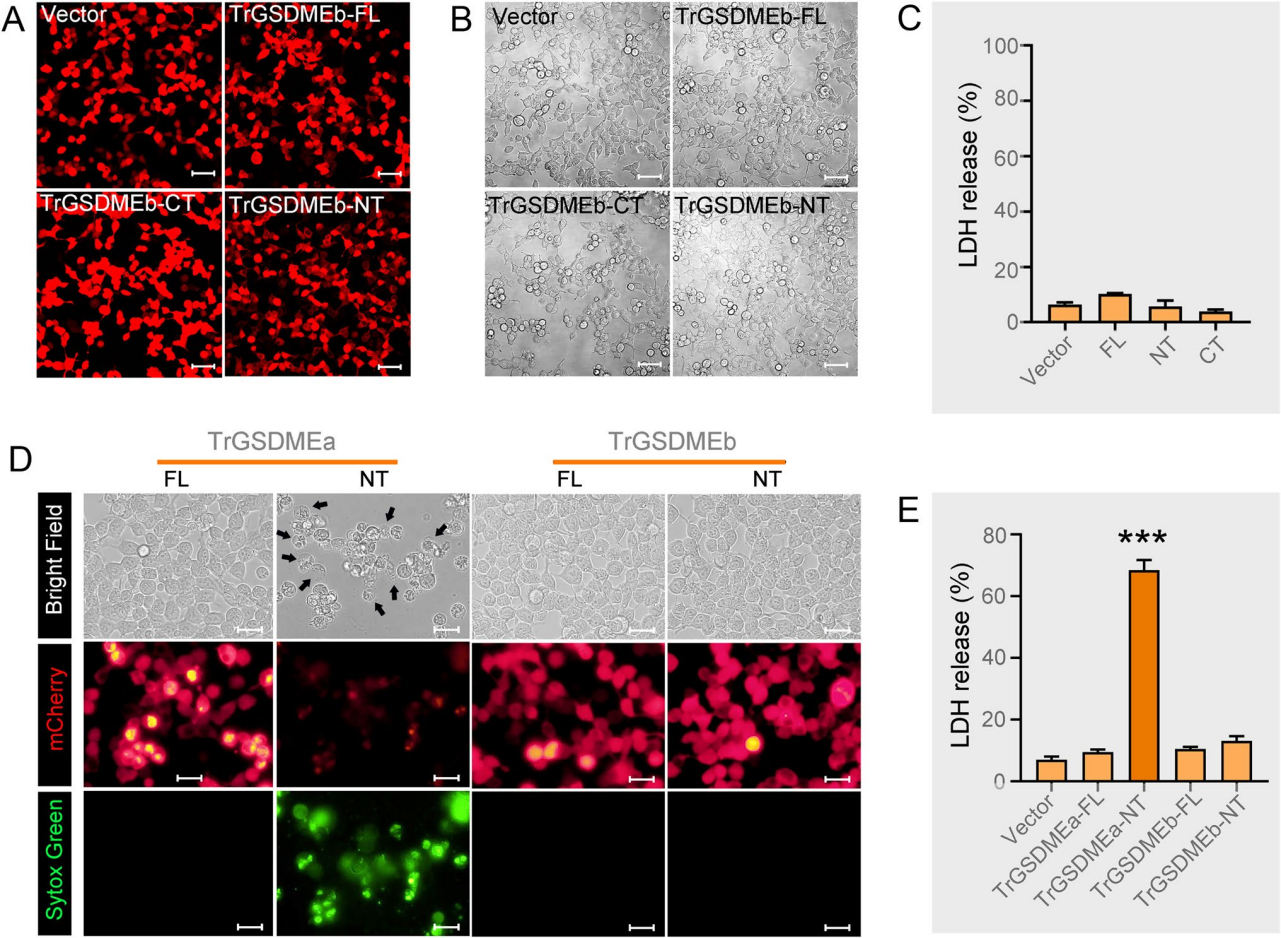
The cytotoxic potential of TrGSDMEb. TrGSDMEb-FL, -NT, and -CT were tagged with mCherry and expressed in HEK293T cells for 24 h. The cells were analyzed for TrGSDME expression (**A**), morphological change (**B**), and LDH release (**C**). Scale bar, 50 μm. **D** HEK293T cells were transfected with mCherry-tagged TrGSDMEa/b–FL/NT/CT for 24 h. The cells were stained with Sytox green and analyzed with microscopy. Representative pyroptotic cells were indicated with black arrows. Scale bar, 30 μm. **E** The LDH release from the above transfected cells of **D** was measured. For panels C and E, values are the means ± SD of triplicate experiments. ***P < 0.001

To examine whether TrGSDMEb possessed pyroptosis-inducing activity, mCherry-tagged full length (FL) or the NT/CT fragments of TrGSDMEb were expressed in HEK293T cells. Microscopy showed that TrGSDMEb-FL, -NT, and -CT were abundantly expressed in the transfected cells (Fig. 2A). No significant difference in morphology or LDH release was observed in the cells expressing TrGSDME-FL or -NT/CT variants (Fig. 2B, C). Sytox Green staining showed that TrGSDMEa-NT expression damaged the cell structure and allowed Sytox Green to enter the cells (Fig. 2D), but TrGSDMEb-NT expression had no apparent effect on the cells (Fig. 2D). A large amount of LDH release was observed in the cells expressing TrGSDMEa-NT but not in the cells expressing TrGSDMEb-NT (Fig. 2E). This indicated that, in contrast to TrGSDMEa, TrGSDMEb was not a typical pyroptosis executioner.

### Bacterial pathogens regulate TrGSDME and TrCASP3/7 expression

**Fig. 3:**
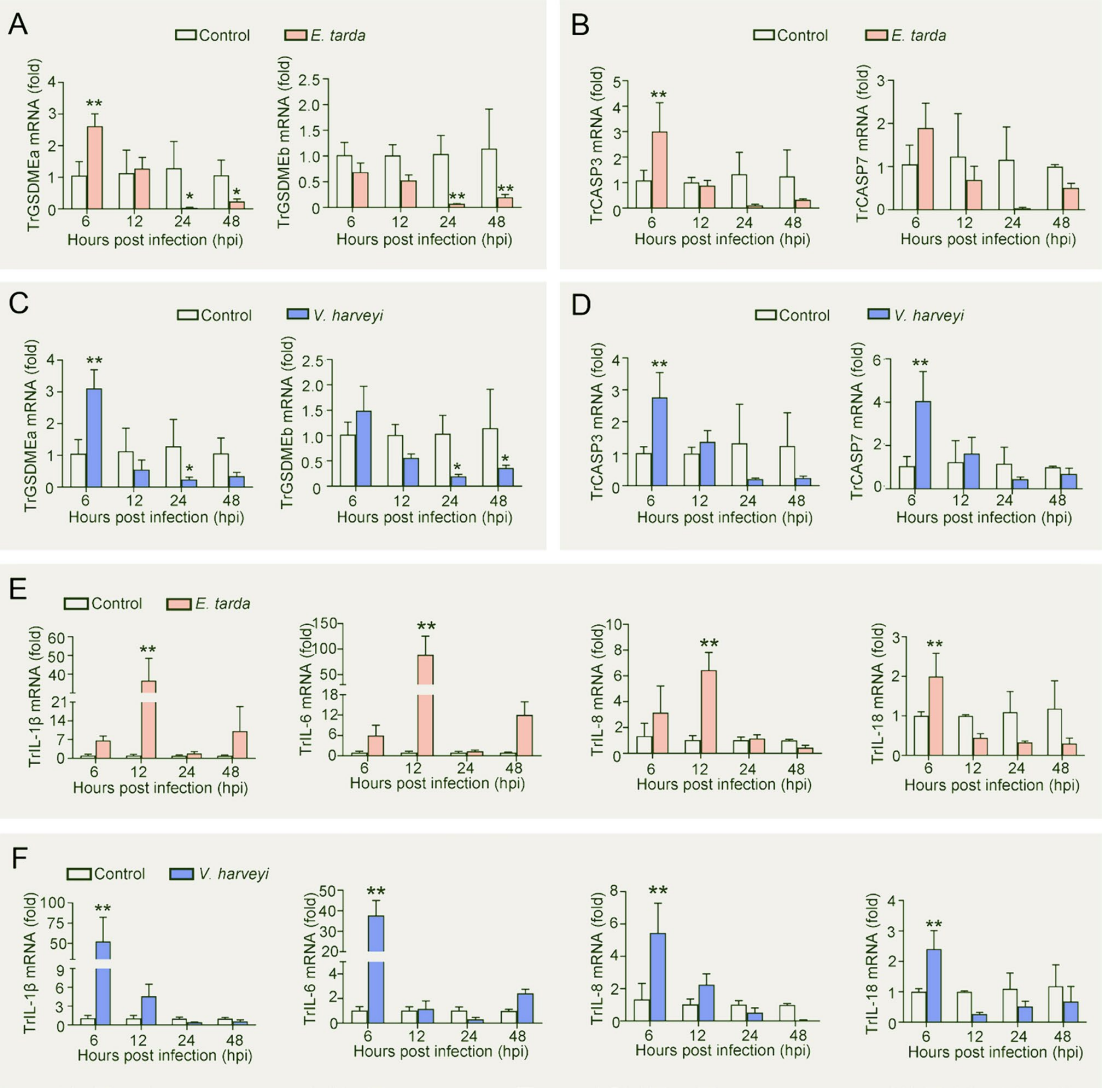
TrGSDME, TrCASP3/7, and inflammatory cytokine expression in pufferfish in response to bacterial infection. Pufferfish were infected with or without (control) *Edwardsiella tarda* for different hours, and the expression of TrGSDMEa/b (**A**) and TrCASP3/7 (**B**) in kidney was determined by qRT-PCR. Pufferfish were infected with *Vibrio harveyi*, and the expression of TrGSDMEa/b (**C**) and TrCASP3/7 (**D**) was determined as above. Pufferfish were infected with or without (control) *E. tarda* (**E**) or *V. harveyi* (**F**), and TrIL-1β, TrIL-6, TrIL-8, and TrIL-18 expression in kidney were determined by qRT-PCR at various hours. For all panels, values are the means ± SD. *n* = 3. **P < 0.01; *P < 0.05

To examine whether pufferfish GSDME participated in pathogen infection, qRT-PCR was used to characterize the expression of GSDME orthologs under different conditions. In the absence of infection, TrGSDMEa and TrGSDMEb exhibited similar expression profiles in the intestine, kidney, muscle, liver, skin, spleen, and gill of pufferfish. The highest expression levels occurred in the gill and the lowest levels in the intestine (Supplementary Fig. S5). When the fish were infected with *E. tarda*, TrGSDMEa expression significantly increased at 6 hpi and decreased at 24 and 48 hpi, while TrGSDMEb expression significantly decreased at 24 and 48 hpi (Fig. 3A). Since TrGSDMEa is cleaved by TrCASP3/7 to induce pyroptosis, the expression of TrCASP3/7 was also examined. TrCASP3/7 expression significantly increased at 6 h post *E. tarda* infection (Fig. 3B). During the infection of *V. harveyi*, TrGSDMEa expression was significantly upregulated at 6 hpi and downregulated at 24 hpi, while TrGSDMEb expression was significantly downregulated at 24 and 48 hpi, and TrCASP3/7 expression was significantly upregulated at 6 hpi (Fig. 3C, D). In addition to TrGSDME and TrCASP3/7, *E. tarda* and *V. harveyi* infection significantly upregulated the expression of the pufferfish inflammatory cytokines IL-1β, IL-6, IL-8, and IL-18 (named TrIL-1β, TrIL-6, TrIL-8, and TrIL-18, respectively) in a time-dependent manner (Fig. 3E, F).

### Bacterial infection activates TrGSDMEa and TrCASP3/7

**Fig. 4:**
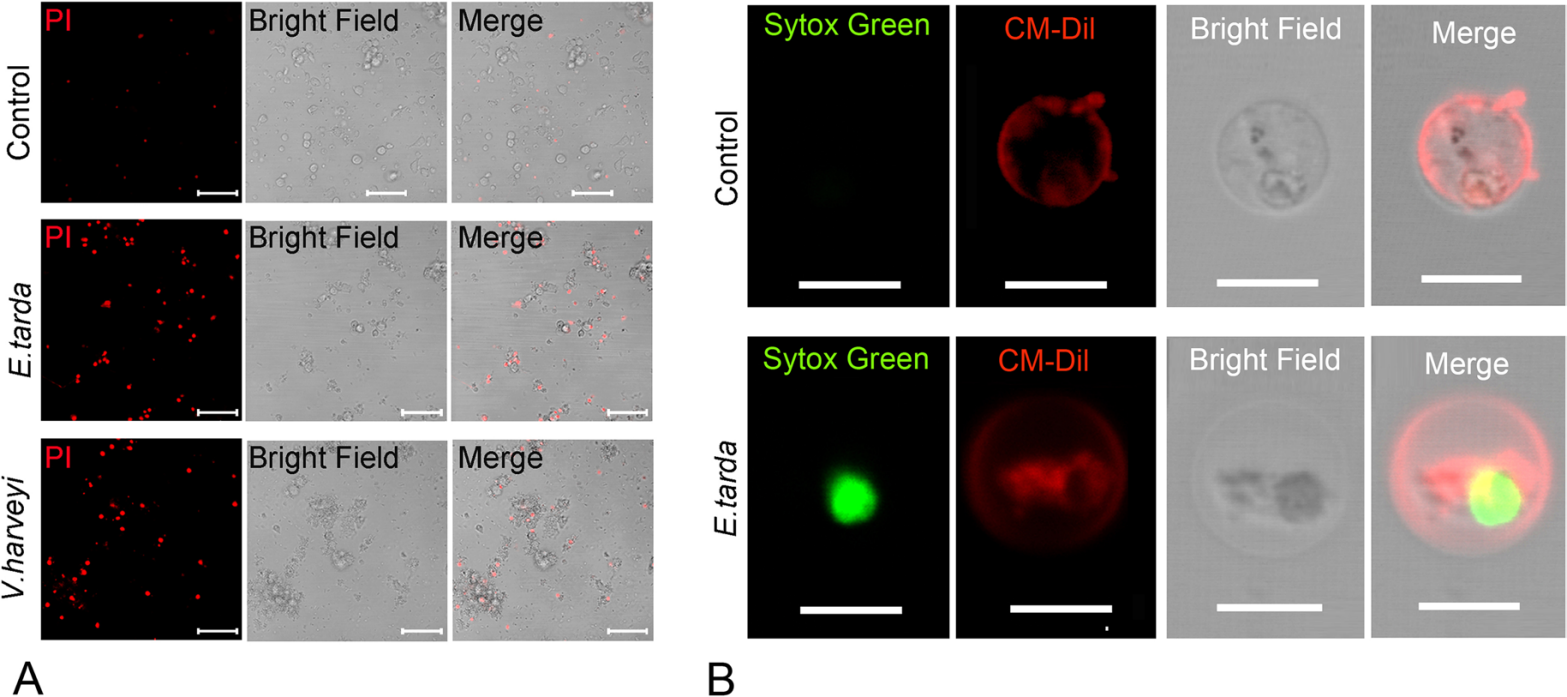
The effect of bacterial infection on the death of pufferfish cells. **A** Pufferfish macrophages were infected with or without (control) *Edwardsiella tarda* or *Vibrio harveyi* for 1 h and then treated with PI. Scale bar, 50 μm. **B** Pufferfish macrophages were incubated with or without (control) *E. tarda* for 3 h and then stained with Sytox green and CM-Dil. Scale bar, 5 μm

**Fig. 5:**
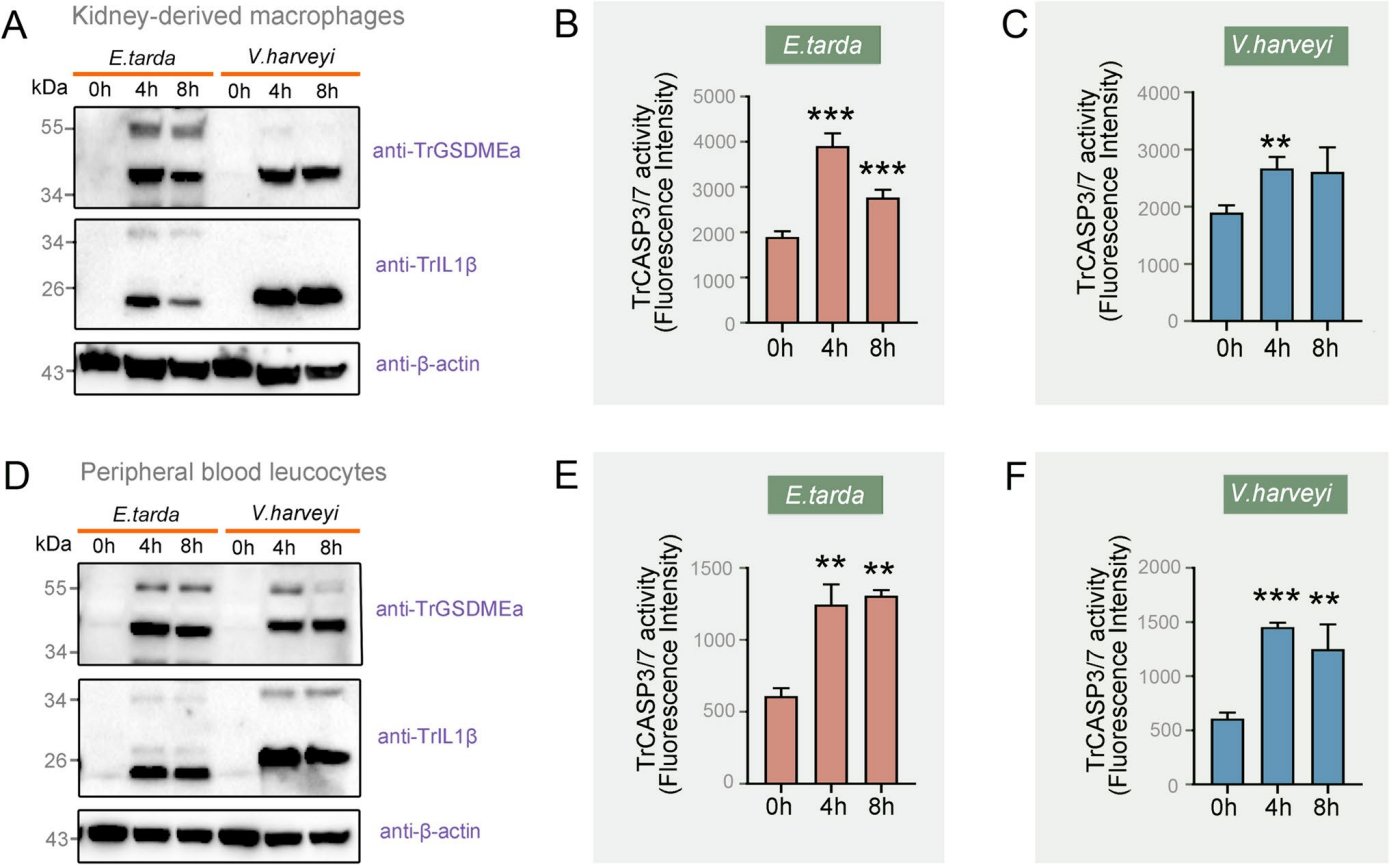
TrGSDMEa and TrCASP3/7 activation in pathogen-infected pufferfish cells. Pufferfish macrophages were incubated with *Edwardsiella tarda* or *Vibrio harveyi* for various hours. The cells and culture supernatant were immunoblotted with antibodies against TrGSDMEa, TrIL-1β, or β-actin (**A**) and assayed for TrCASP3/7 activity (**B, C**). Pufferfish peripheral blood leucocytes were infected with *E. tarda* or *V. harveyi* and then immunoblotted as above (**D**) and assayed for TrCASP3/7 activity (**E**, **F**). For panels (**B**, **C**, **E**, **F**), values are shown as means ± SD of three experimental replicates. ***P < 0.001; **P < 0.01

To determine the involvement of TrGSDME in bacterial infection, we examined the cell death of pufferfish kidney-derived macrophages after bacterial infection. Microscopy revealed that the macrophages treated with *E. tarda* or *V. harveyi*, two common bacterial pathogens to fish, were highly susceptible to PI staining (Fig. 4A), indicating cellular membrane disruption. The infected cells were swollen up and stained by Sytox Green, but maintained intact nuclei (Fig. 4B). The cells exhibited strong induction and cleavage of TrGSDMEa, accompanied with the production of mature IL-1β (Fig. 5A). In agreement with the massive activation of TrGSDMEa, the activation of TrCASP3/7 in the infected cells also showed a time-dependent pattern (Fig. 5B, C). Similar results were obtained with the peripheral blood leucocytes infected with bacteria (Fig. 5D–F).

### TrCASP3/7 are vital to pufferfish defense against bacterial pathogens

**Fig. 6:**
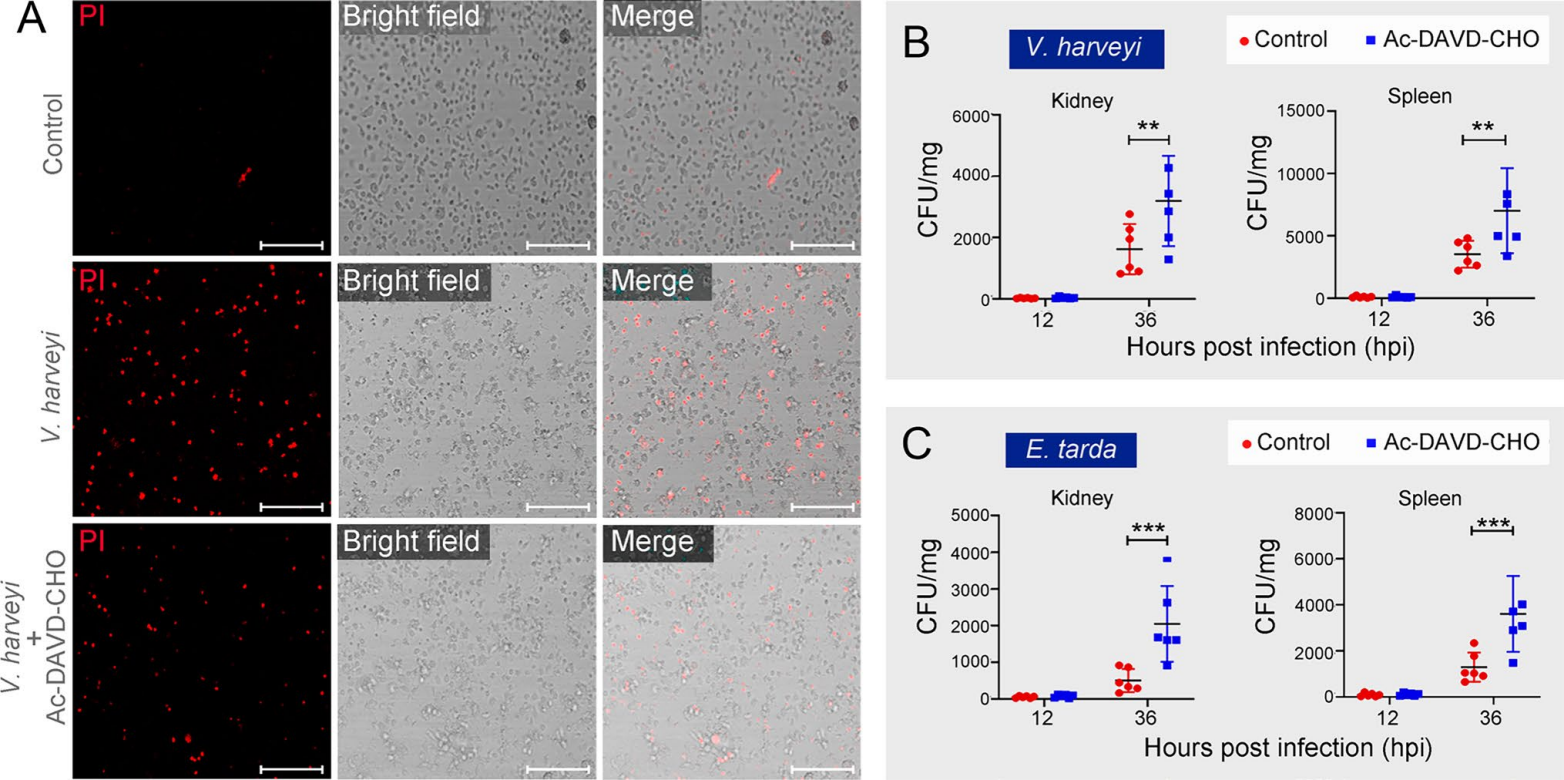
The effect of TrCASP3/7 inhibitor on bacterial infection in pufferfish. **A** Pufferfish macrophages were infected with or without (control) *Vibrio harveyi* in the presence or absence of AC-DAVD-CHO for 1 h. The cells were stained with PI and observed with a microscope. Bar size, 50 μm. Pufferfish were infected with *V. harveyi* (**B**) or *Edwardsiella tarda* (**C**) in the presence or absence (control) of AC-DAVD-CHO, and bacterial loads (shown as colony-forming unit, CFU) in kidney and spleen were determined at different hours. Values are the means ± SD. *n* = 6. ***P < 0.001; **P < 0.01

To explore the role of the TrCASP3/7-TrGSDMEa axis in antibacterial infection, the tetrapeptide Ac-DAVD-CHO was used as an inhibitor of TrCASP3/7 to block TrGSDMEa cleavage by TrCASP3/7. Pufferfish macrophages treated with Ac-DAVD-CHO exhibited reduced cell death after *V. harveyi* infection (Fig. 6A). When the fish were infected with *V. harveyi* in the presence of Ac-DAVD-CHO, the bacterial loads increased drastically in spleen and kidney (Fig. 6B). Similarly, the presence of Ac-DAVD-CHO significantly increased the dissemination of *E. tarda* in pufferfish kidney and spleen (Fig. 6C).

### Identification of key residues in teleost GSDMEa that are essential to pyroptosis

**Fig. 7:**
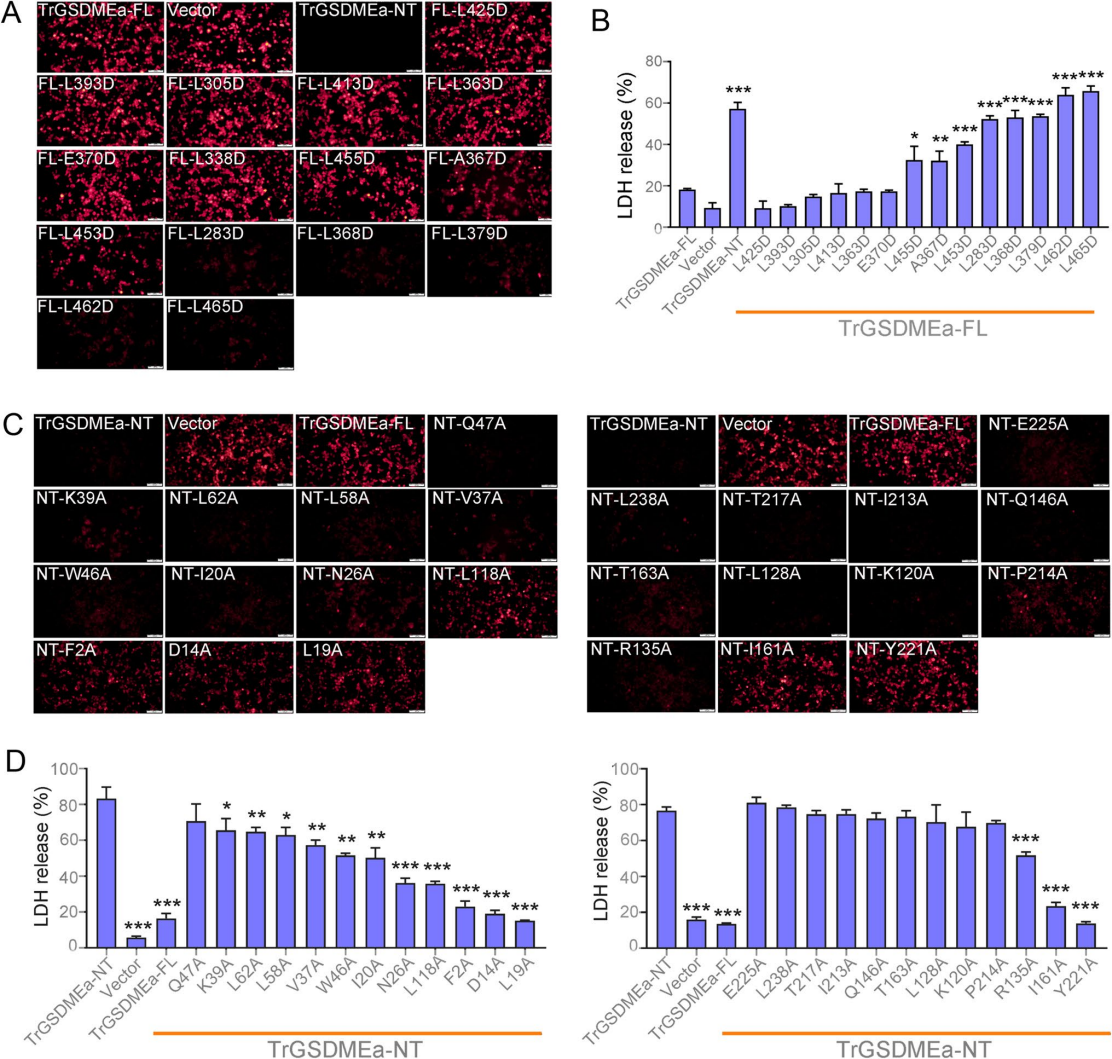
The pyroptosis activity of TrGSDMEa variants. HEK293T cells were transfected with mCherry-tagged full-length (FL) TrGSDMEa or its mutants for 24 h and then observed with microscope (**A**) and measured for LDH release (**B**). HEK293T cells transfected with TrGSDMEa-NT was used as a positive control. HEK293T cells were transfected with mCherry-tagged TrGSDMEa NT domain or mutated NT domain for 24 h and then observed with a microscope (**C**) and measured for LDH release (**D**). Values in **B** and **D** are the means ± SD of triplicate experiments. ***P < 0.001; **P < 0.01; *P < 0.05. Scale bar in **A** and **C**, 100 μm

Zebrafish and turbot GSDMEa with pyroptotic activity have been reported (Wang et al. 2017; Xu et al. 2022a). Sequence alignment showed that the GSDMEa of zebrafish, turbot, and pufferfish shared conserved residues with each other and with human/mouse GSDME (Supplementary Fig. S6). Twenty-four conserved residues in the NT domain and fourteen conserved residues in the CT domain were selected for mutation analysis (Supplementary Fig. S6). The TrGSDMEa mutants with L283D, A367D, L368D, L379D, L453D, L455D, L462D, and L465D substitutions in the CT domain lost the CT inhibitory function and exhibited spontaneous pyroptosis-inducing activity. This resulted in LDH release from the dying cells (Fig. 7A, B), whereas the mutants with F2A, D14A, L19A, N26A, L118A, R135A, I161A, and Y221A substitutions in the NT domain had significantly impaired pyroptosis induction (Fig. 7C, D). The mutants of K39A, L62A, L58A, V37A, W46A, and I20A were moderately, though significantly, defective in executing pyroptosis (Fig. 7C, D). Other mutants (Q47A, K120A, L128A, Q146A, T163A, P214A, T217A, E225A, L238A, L305D, L363D, E370D, L393D, and L425D) exhibited no apparent change in membrane-perforation or auto-inhibition activity. Together, these results indicated that F2, D14, L19, N26, L118, R135, I161, and Y221 in the NT domain, and the L283, A367, L368, L379, L453, L455, L462, and L465 in the CT domain were key residues for pore formation and auto-inhibition, respectively. All the above identified key residues crucial for TrGSDMEa-NT-mediated pyroptosis are conserved in TrGSDMEb-NT (Supplementary Fig. S3).

## Discussion

During GSDM-mediated pyroptosis, proteolytic cleavage of the full-length GSDM to release the lipophilic NT domain is required. In mammals, GSDME is cleaved by the apoptotic CASP3 to liberate the NT fragment, which then switches cell death from apoptosis to pyroptosis (Rogers et al. 2017; Wang et al. 2017). In contrast to humans, which have one GSDME, teleost generally possess two GSDME orthologs, GSDMEa and GSDMEb (Yuan et al. 2022). We have demonstrated that TrGSDMEa is cleaved by CASP3/7 at the DIVD site to produce the pyroptotic NT domain (Xu et al. 2024). In the present study, we found that TrGSDMEb was cleaved by CASP1/3/6/7/8 at the FEVD motif to generate an NT fragment, which, however, could not induce pyroptosis. TrGSDMEb shares a similar overall structure with TrGSDMEa and possesses all the eight key residues involved in TrGSDMEa-mediated pyroptosis. These results suggest the possibility that it is some minor structural changes that cause the dramatic functional difference between TrGSDMEb and TrGSDMEa. Indeed, we observed differences in some small helical structures between TrGSDMEb and TrGSDMEa, which may prove to influence the function of the proteins. In addition, it is possible that the key residues identified in this study represent but a fraction of the total amount of residues that are essential to pyroptosis, and other yet to be discovered key residues may be different between TrGSDMEb and TrGSDMEa, therefore contributing to the functional difference between TrGSDMEb and TrGSDMEa. The inability of TrGSDMEb to induce pyroptosis is an observation similar to that reported in turbot and zebrafish, which are the fish species with documented comparative studies of the pyroptosis activities of GSDMEa and GSDMEb. In turbot, GSDMEa is cleaved by CASP3/7 to release the pyroptotic NT domain, while GSDMEb is cleaved by CASP8 to produce an NT fragment without pyroptosis activity (Xu et al. 2022a). In zebrafish, compared to the GSDMEa-NT domain, the GSDMEb-NT domain only weakly induces pyroptosis and LDH release (Chen et al. 2021). These results, together with our observation of TrGSDMEb in this study, suggest that in teleost, GSDMEa and GSDMEb have likely differentiated into functionally distinct molecules. While GSDMEa retains the ability to execute pyroptosis, GSDMEb may have lost pyroptosis-inducing capacity but gained other functionality. The biological function of fish GSDMEb remains to be investigated in future studies.

**Fig. 8:**
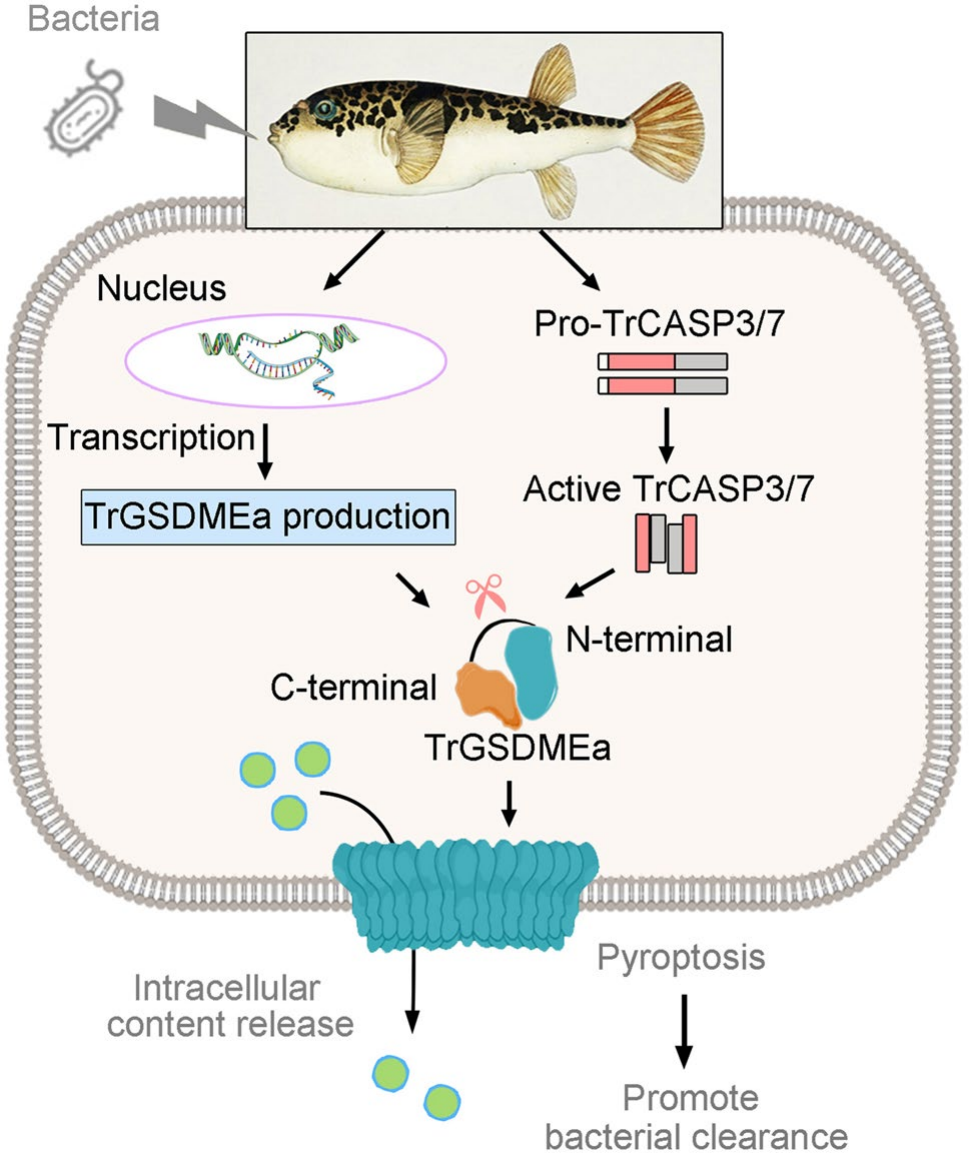
A proposed model of the anti-bacterial effect of TrGSDMEa-mediated pyroptosis in pufferfish. Bacterial infection causes the production of TrGSDMEa and the activation of TrCASP3/7. Activated TrCASP3/7 then cleave TrGSDMEa to trigger pyroptosis, which leads to intracellular content release and effective bacterial clearance

The regulation and function of GSDM in bacterial infection have been extensively studied in mammals (Booty and Bryant 2022; Man et al. 2017). In humans, there are six members of GSDM (GSDMA to E, and PJVK). Among these, GSDMD is well known for its role in antibacterial infection (Magnani et al. 2022; Wang et al. 2019; Zhu et al. 2018). In contrast, teleost lack GSDMA to D and carry out pyroptosis solely through GSDME (Hofmann 2020; Wang et al. 2017). In turbot, GSDMEa was activated during bacterial infection and promoted bacterial clearance (Xu et al. 2022a). In common carp, GSDMEb could promote IL-1β secretion and resistance against bacterial colonization (Zhao et al. 2023). In pufferfish, both *E. tarda* and *V. harveyi* infection induced TrGSDMEa expression at the mRNA and protein levels, suggesting involvement of TrGSDMEa in bacterial defense. The infected cells exhibited pyroptotic cell death, which was consistent with upregulated CASP3 activity and TrGSDMEa cleavage. In mammals, GSDMD-mediated pyroptosis is activated by CASP1 cleavage and accompanied with the release of mature IL-1β, as a result of CASP1 cleavage of the precursor IL-1β (Broz et al. 2020; Keller et al. 2008; Mantovani et al. 2019; van de Veerdonk et al. 2011). In fish, the association between IL-1β maturation and pyroptosis is unclear. In the present study, we found that *E. tarda* and *V. harveyi* infection induced pufferfish cells to undergo pyroptosis, and during this process, both IL-1β production and maturation were increased. This result suggested that in pufferfish, IL-1β may be activated by CASP3/7 cleavage. In line with these observations, in vivo infection with *E. tarda* and *V. harveyi* significantly upregulated the expression of not only TrGSDMEa and TrCASP3/7, which execute pyroptosis, but also the cytokines of IL-1β, IL-6, IL-8, and IL-18 in pufferfish, suggesting that cytokine production was likely a result of pyroptosis-induced immune response. The production of these inflammatory cytokines aids in infection clearance. Blocking TrCASP3/7 activity significantly inhibited the death of infected cells and promoted the dissemination of *E. tarda* and *V. harveyi* in fish tissues. These results highlighted the important role of TrGSDMEa-mediated pyroptosis in antibacterial immunity (Fig. 8).

The membrane pore-forming activity of GSDM-NT domain is normally inhibited by the CT domain (Ding et al. 2016; Rogers and Alnemri 2019). With the lack of higher ordered structures, the working mechanisms of the NT and CT domains of GSDME remain to be explored. In the present work, a scanning mutagenesis identified the residues of L283, A367, L368, L379, L453, L455, L462, and L465 as essential to the auto-inhibition activity of the TrGSDMEa-CT domain. Of these residues, L465 corresponds to human GSDME L491, which participates in the auto-inhibition of the CT domain (Yuan et al. 2022). In contrast, mutation of the L451 in human GSDME significantly reduced the inhibitory effect of the CT domain (Yuan et al. 2022), while mutation of the corresponding residue (L425) in TrGSDMEa had no apparent effect on auto-inhibition. These results suggest that the structure of the teleost GSDME-CT domain may differ from that of human GSDME-CT. Previous research demonstrated that GSDME-CT domain contributed to the difference in the cleavability of teleost and human GSDME by CASP7 (Xu et al. 2024). In addition to the essential CT residues, we also identified F2, D14, L19, N26A, L118, R135, I161, and Y221 as essential NT residues required for the pore-forming activity of TrGSDMEa. Of these residues, F2 is highly conserved in both invertebrate and vertebrate GSDM (Chen et al. 2023; Jiang et al. 2020; Qin et al. 2023; Rogers et al. 2017). Mutation of F2 significantly reduced the cell death-inducing capacity of TrGSDMEa-NT. This result was consistent with the previous reports demonstrating that F2A mutation significantly decreased the killing activity of human and abalone GSDME (Qin et al. 2023; Rogers et al. 2017). D14, L19, and N26 are located in the first 56 residues of GSDME, which are involved in membrane targeting and penetration (Feng et al. 2018; Rogers et al. 2017). I161 is located in the β7 region of the TrGSDMEa-NT domain. This β7 strand, together with α4 and β8, stretches out into the second transmembrane β-hairpin in GSDMA3 (Ruan et al. 2018). Therefore, mutation of I161 might interfere with formation of the transmembrane β-hairpin, thereby affecting pyroptosis. R135 is highly conserved in mammalian GSDM, but mutation of the corresponding residue in mouse GSDMA3 (R132) barely compromised pore formation (Ruan et al. 2018). Together, these results suggest that different mechanisms of pore formation and auto-inhibition exist in teleost and mammalian GSDME.

### Supplementary Information

Below is the link to the electronic supplementary material.

Supplementary file1 (DOCX 1878 KB)

## Data Availability

All data in this study can be accessed in the paper or the Supplementary materials.
