# Supplementary material for: Pufferfish gasdermin Ea is a significant player in the defense against bacterial pathogens
Source: Mar Life Sci Technol. 2024 Jun 28;6(3):462–74. doi: 10.1007/s42995-024-00237-x (PMC11358365; doi:10.1007/s42995-024-00237-x)
Supplement: Supplementary file 1 — Supplementary file1 (DOCX 1878 KB) [file 42995_2024_237_MOESM1_ESM.docx]

**Supplementary materials**

**The file include:**

Supplementary Table S1. Primers used in this study.

Supplementary Fig. S1. Phylogenetic analysis of TrGSDMEa/b.

Supplementary Fig. S2. Preparation of recombinant caspases.

Supplementary Fig. S3. Sequence alignment of TrGSDMEa-NT and TrGSDMEb-NT.

Supplementary Fig. S4. Comparison of the predicted 3D structures of TrGSDMEa-NT and TrGSDMEb-NT.

Supplementary Fig. S5. The expression of TrGSDME in pufferfish tissues.

Supplementary Fig. S6. Sequence analysis of teleost GSDMEa.

**Supplementary Table S1. Primers used in this study.**

| Primer | Sequence (5’-3’) |
| --- | --- |
| Primers for gene cloning  TrGSDMEb forward  TrGSDMEb reverse  TrCASP1 forward  TrCASP1 reverse  TrCASP6 forward  TrCASP6 reverse  TrCASP8 forward  TrCASP8 reverse  TrIL-1β forward  TrIL-1β reverse  TrIL-18 forward  TrIL-18 reverse  TrIL-18 forward  TrIL-18 reverse  Primers for recombinant protein expression  TrGSDMEb forward  TrGSDMEb reverse  TrCASP1 forward  TrCASP1 reverse  TrCASP6 forward  TrCASP6 reverse  TrGSDMEb-D245R forward  TrGSDMEb-D248R reverse  TrIL-1β forward  TrIL-1β reverse  TrIL-18 forward  TrIL-18 reverse  Primers for gene overexpression  TrGSDMEb-FL forward  TrGSDMEb-FL reverse  TrGSDMEb-NT forward  TrGSDMEb-NT reverse  TrGSDMEb-CT forward  TrGSDMEb-CT reverse  TrGSDMEa-F2A forward  TrGSDMEa-F2A reverse  TrGSDMEa-D14A forward  TrGSDMEa-D14A reverse  TrGSDMEa-L19A forward  TrGSDMEa-L19A reverse  TrGSDMEa-I20A forward  TrGSDMEa-I20A reverse  TrGSDMEa-N26A forward  TrGSDMEa-N26A reverse  TrGSDMEa-V37A forward  TrGSDMEa-V37A reverse  TrGSDMEa-K39A forward  TrGSDMEa-K39A reverse  TrGSDMEa-W46A forward  TrGSDMEa-W46A reverse  TrGSDMEa-Q47A forward  TrGSDMEa-Q47A reverse  TrGSDMEa-L58A forward  TrGSDMEa-L58A reverse  TrGSDMEa-L62A forward  TrGSDMEa-L62A reverse  TrGSDMEa-L118A forward  TrGSDMEa-L118A reverse  TrGSDMEa-K120A forward  TrGSDMEa-K120A reverse  TrGSDMEa-R135A forward  TrGSDMEa-R135A reverse  TrGSDMEa-Q146A forward  TrGSDMEa-Q146A reverse  TrGSDMEa-I161A forward  TrGSDMEa-I161A reverse  TrGSDMEa-T163A forward  TrGSDMEa-T163A reverse  TrGSDMEa-I213A forward  TrGSDMEa-I213A reverse  TrGSDMEa-P214A forward  TrGSDMEa-P214A reverse  TrGSDMEa-T217A forward  TrGSDMEa-T217A reverse  TrGSDMEa-Y221A forward  TrGSDMEa-Y221A reverse  TrGSDMEa-E225A forward  TrGSDMEa-E225A reverse  TrGSDMEa-L238A forward  TrGSDMEa-L238A reverse  TrGSDMEa-L283D forward  TrGSDMEa-L283D reverse  TrGSDMEa-I298D forward  TrGSDMEa-I298D reverse  TrGSDMEa-L338D forward  TrGSDMEa-L338D reverse  TrGSDMEa-L363D forward  TrGSDMEa-L363D reverse  TrGSDMEa-A367D forward  TrGSDMEa-A367D reverse  TrGSDMEa-L368D forward  TrGSDMEa-L368D reverse  TrGSDMEa-E370D forward  TrGSDMEa-E370D reverse  TrGSDMEa-L379D forward  TrGSDMEa-L379D reverse  TrGSDMEa-L393D forward  TrGSDMEa-L393D reverse  TrGSDMEa-L413D forward  TrGSDMEa-L413D reverse  TrGSDMEa-L425D forward  TrGSDMEa-L425D reverse  TrGSDMEa-L453D forward  TrGSDMEa-L453D reverse  TrGSDMEa-L455D forward  TrGSDMEa-L455D reverse  TrGSDMEa-L462D forward  TrGSDMEa-L462D reverse  TrGSDMEa-L465D forward  TrGSDMEa-L465D reverse | ATGTTCGCCGCAGCTACC  CTGAGCCAGTGAGGCGAG  GCAGTGCTGGGTCTGTCC  GATGAGGCCTGGGTAGAAGTA  ATGTCAAACAAGACAGAAGACATTC  TTACTTCTTTGGGCGGAAGT  AATGCACAGAACTCAGCAATGA  CAGAACAGGATGAGGAGTGTTG  ATGGAATCTCAGATGAAATCCAACG  TTACATCTCTCCCTCACACGTGG  ATGGCAGCTAACAATGGCA  CTAAGACATGGTGACGTAGCAGG  ATGGCAGCTAACAATGGCA  AGACATGGTGACGTAGCAGGTC  TAAGAAGGAGATATACATATGATGTTC  GCCGCAGCTACC  GTGGTGGTGGTGGTGCTCGAGCTGAGC  CAGTGAGGCGAG  TAAGAAGGAGATATACATATGGCAGTGC  TGGGTCTGTCC  GTGGTGGTGGTGGTGCTCGAGGATGAGG  CCTGGGTAGAAGTA  TAAGAAGGAGATATACATATGATGTCAA  ACAAGACAGAAGACATTC  GTGGTGGTGGTGGTGCTCGAGTTACTTCT  TTGGGCGGAAGT  GTTTTGAAGTGGCAGGCCCCCCCCGGAC  AAC  GTTGTCCGGGGGGGGCCTGCCACTTCAA  AAC  GCCATGGCTGATATCGGATCCATGGAAT  CTCAGATGAAATCCAACG  CTCGAGTGCGGCCGCAAGCTTTTACATCT  CTCCCTCACACGTGG  TAAGAAGGAGATATACATATGATGGCAG  CTAACAATGGCA  TCAGATCTCGAGCTCAAGCTTATGTTCGCC  GCAGCTACC  CATGGTGGCGACCGGTGGATCCTGAGCCA  GTGAGGCGAG  TCAGATCTCGAGCTCAAGCTTATGTTCGCC  GCAGCTACC  CATGGTGGCGACCGGTGGATCGTCCACTT  CAAAACCTCCCTTAA  TCAGATCTCGAGCTCAAGCTTATGGGCCCC  CCCCGGA  CATGGTGGCGACCGGTGGATCCTGAGCCA  GTGAGGCGAG  GCTCAAGCTTATGGCTTCCAAGGCCACGG  TCGAGTTCGAATACCGAAGGTTCCGGTGC  GTCCATCAGATTGCCCCAGAGGGAAG  AGGTAGTCTAACGGGGTCTCCCTTCG  ATTGACCCAGAGGGAAGCGCTATCCCCGT  CTCCCTTCGCGATAGGGGCAGAGGTCTC  CAGAGGGAAGCCTCGCACCCGTCTCCAG  GTCTCCCTTCGGAGCGTGGGCAGAGGTCTC  CCGTCTCCAGAGTTGCTGACTCCAAGAAGC  CAGAGGTCTCAACGACTGAGGTTCTTCG  TCCATGGCACTGGCTGTCAAGCGCAAC  AGGTACCGTGACCGACAGTTCGCGTTG  GGCACTGGTTGTCGCGCGCAACCGCAGATG  CGTGACCAACAGCGCGCGTTGGCGTCTACC  ACCGCAGATGGTTCGCACAGAGGCCCAAG  TTGGCGTCTACCAAGCGTGTCTCCGGGTT  ACCGCAGATGGTTCTGGGCGAGGCCCAAG  TACTAC  GGCGTCTACCAAGACCCGCTCCGGGTTCAT  GATGG  CGACAGATTTCACCGCAAGCCACCTGCTG  CTGCAGCAGGTGGCTTGCGGTGAAATCTG  TTTCACCCTGAGCCACCTGGCACAGGGCGA  CAAAGACCTC  AAGTGGGACTCGGTGGACCGTGTCCCGCT  GTTTCTGGAGG  CGGTCATTTTTTGGCAAGGCGAAGAAAGAA  GAACTGG  CCAGTAAAAAACCGTTCCGCTTCTTTCTTC  TTGACCT  TTTGGCAAGCTGAAGGCAGAAGAACTGG  AACCGTTCGACTTCCGTCTTCTTGACCT  AGGACTCCAAAGACGCGCAGGTGGACAT  CCTGAGGTTTCTGCGCGTCCACCTGTAC  ACATGCTGGTGCAGGCGCTGAAGAGGCAA  TGTACGACCACGTCCGCGACTTCTCCGTT  GGTGAAGGAGCGGGCTATCACCACAAGC  CACTTCCTCGCCCGATAGTGGTGTTCGA  GAGCGGATTATCGCCACAAGCTCCTG  CCTCGCCTAATAGCGGTGTTCGAGGA  AGTGACATCTCCCTGGAGGCACCACCC  GGTACGGTCATTG  GTCACTGTAGAGGGACCTCCGTGGTGG  GCCATGCCAGTAA  ACATCTCCCTGGAGATCGCACCCGGTA  CGGTCATTG  CTGTAGAGGGACCTCTAGCGTGGGCCA  TGCCAGTAA  GAGATCCCACCCGGTGCAGTCATTGCG  TACAG  TCTAGGGTGGGCCACGTCAGTAACGCA  TGTCA  GTACGGTCATTGCGGCAAGTGTCCTCG  AGCTG  CCATGCCAGTAACGCCGTTCACAGGAG  CTCGA  ACAGTGTCCTCGCGCTGGAGATCAG  TGTCACAGGAGCGCGACCTCTAGTC  AGTATGGTATATGCGCGCAGCCTGGT  GCC  CATACCATATACGCGCGTCGGACCAC  GGT  CATGCTCTTGCACAGGACCCCAAATC  GACCCG  TACGAGAACGTGTCCTGGGGTTTAGC  TGGGCT  AGGGACTGCAGGACGACATGATTGA  CAGGAG  TTCCCTGACGTCCTGCTGTACTAAC  TGTCCT  GTTCGTGTCAGCCATAGACAAGCAGCTG  GGTTTGG  CAAGCACAGTCGGTATCTGTTCGTCGAC  CCAAACC  TGGAAGCAGTCCATGACCTGGTCAGCGC  TCTG  TACCTTCGTCAGGTACTGGACCAGTCGC  GAGA  ATCTGCTGGTCAGCGATCTGGAGGAGCTG  AGACGACCAGTCGCTAGACCTCCTCGACG  TCTGCTGGTCAGCGCTGATGAGGAGCTGC  CAGATG  AGACGACCAGTCGCGACTACTCCTCGACG  GTCTAC  GCGCTCTGGAGGATCTGCCAGATGAAAC  TCGCGAGACCTCCTAGACGGTCTACTTT  GAAACCTTGGACCTGGACAGCAGGAGCA  CTCC  TTTGGAACCTGGACCTGTCGTCCTCGTG  AGGA  TCTGGAAGCTTTTGACAACGACATGTCCA  GTATTAGAGAGAG  AAGACCTTCGAAAACTGTTGCTGTACAGG  TCATAATCTCTCT  GCCTTCCAGTGCTCGACCAGCAGAACCAGA  TCGGAAGGTCACGAGCTGGTCGTCTTGGTC  AGCTGGCAGAACAGGATCTCACCACCGCCA  GTCGACCGTCTTGTCCTAGAGTGGTGGCGG  GGGCAGAACTCTACATGGACCTGCTCTATC  TCAGTG  CCGTCTTGAGATGTACCTGGACGAGATAGA  GTCACA  AACTCTACATGCTGCTGGACTATCTCAGTG  TACAC  TGAGATGTACGACGACCTGATAGAGTCACA  TGTGC  TCTCAGTGTACACGGAGACTTGCTGCTTGCA  AAGTC  TAGAGTCACATGTGCCTCTGAACGACGAAC  GTTTCA  ACACGGATTGTTGCTGGATGCAAAGTCTGA  AACGG  ATGTGCCTAACAACGACCTACGTTTCAGAC  TTTGC |

**Supplementary Fig. S1. Phylogenetic analysis of TrGSDMEa/b.** The phylogenetic tree is constructed using 17 fish GSDMEa (red) and 67 fish GSDMEb (blue) collected from NCBI orthologs. The TrGSDMEa and TrGSDMEb used in this study are marked with red and blue stars, respectively.


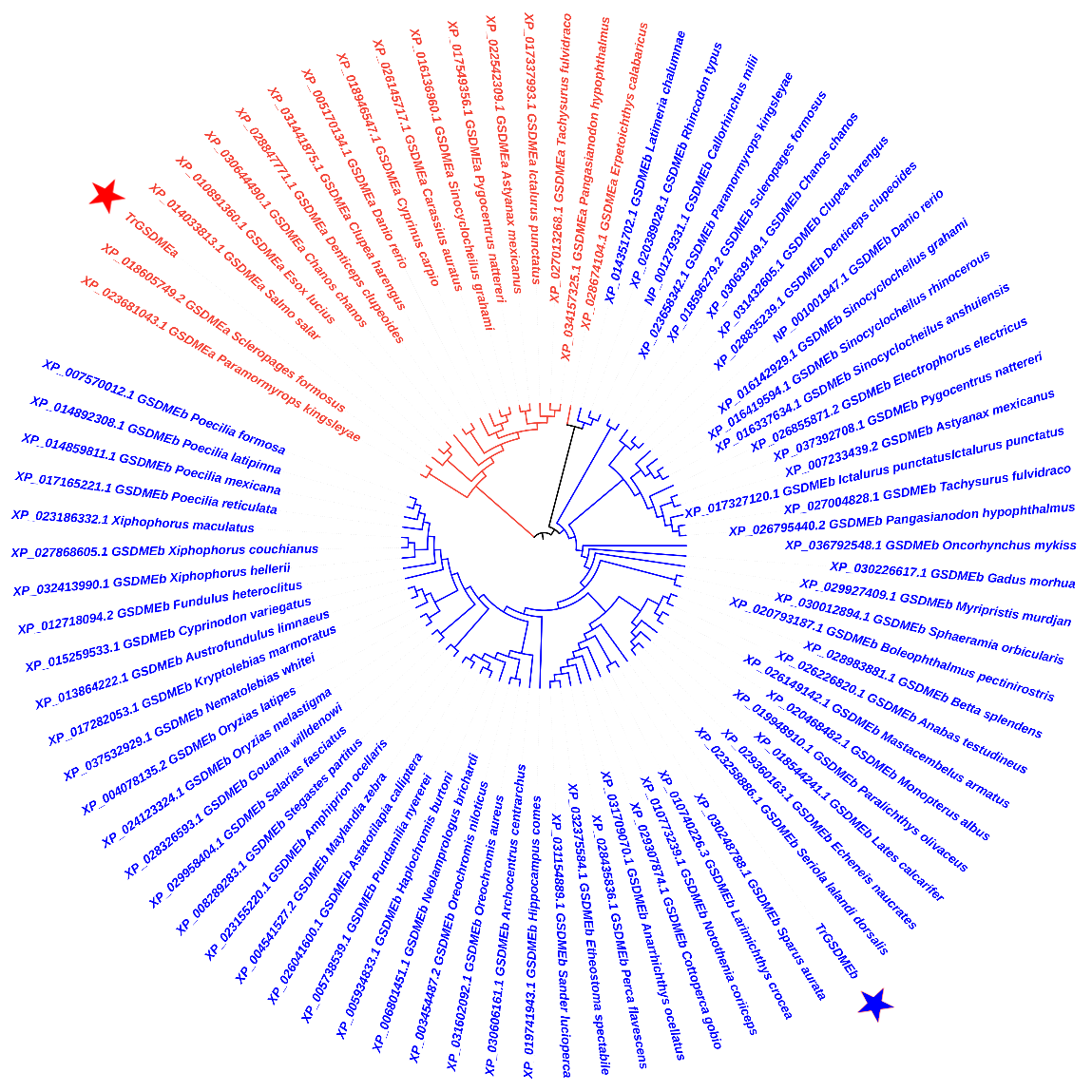


**Supplementary Fig. S2. Preparation of recombinant caspases.** SDS-PAGE analysis of purified TrCASP1 **(A)**, TrCASP6 **(B)**, and TrCASP8 **(C)**.


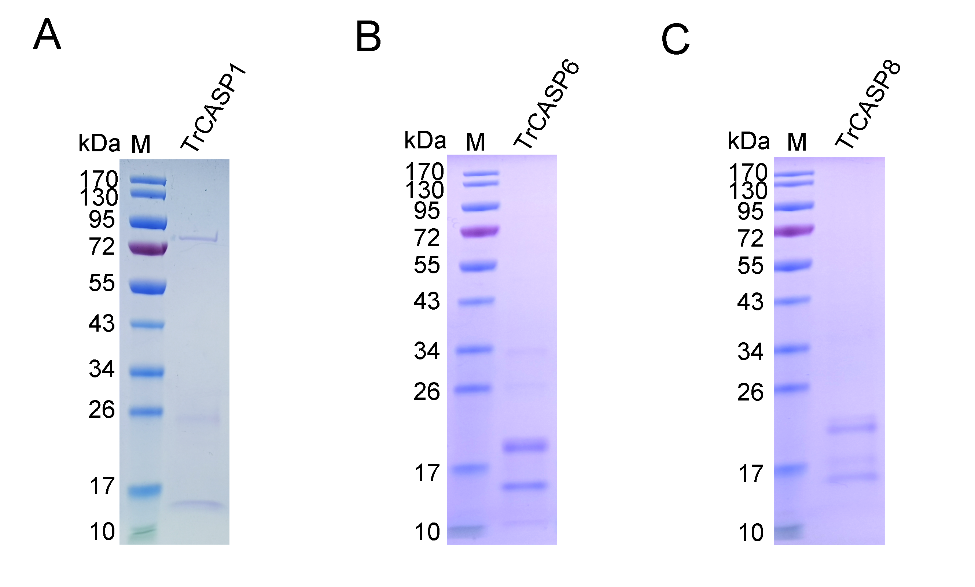


**Supplementary Fig. S3.** **Sequence alignment of TrGSDMEa-NT and TrGSDMEb-NT.** Dots denote gaps introduced for maximum matching. The consensus residues are shaded in black. The residues crucial for TrGSDMEa-mediated pyroptosis are indicated with blue arrows.


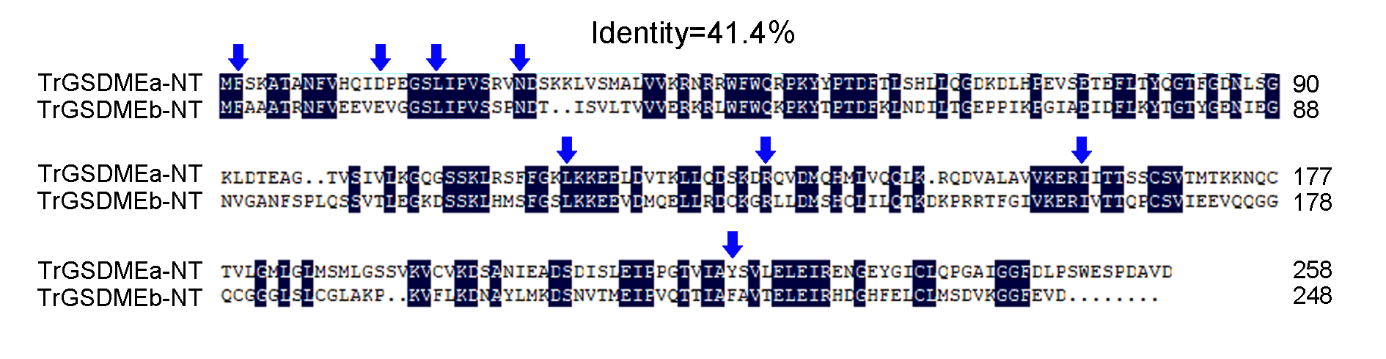


**Supplementary Fig. S4.** **Comparison of the predicted 3D structures of TrGSDMEa-NT and TrGSDMEb-NT.** The N-terminal domains of TrGSDMEa and TrGSDMEb were generated by Robetta, and are shown in red and green, respectively.

**
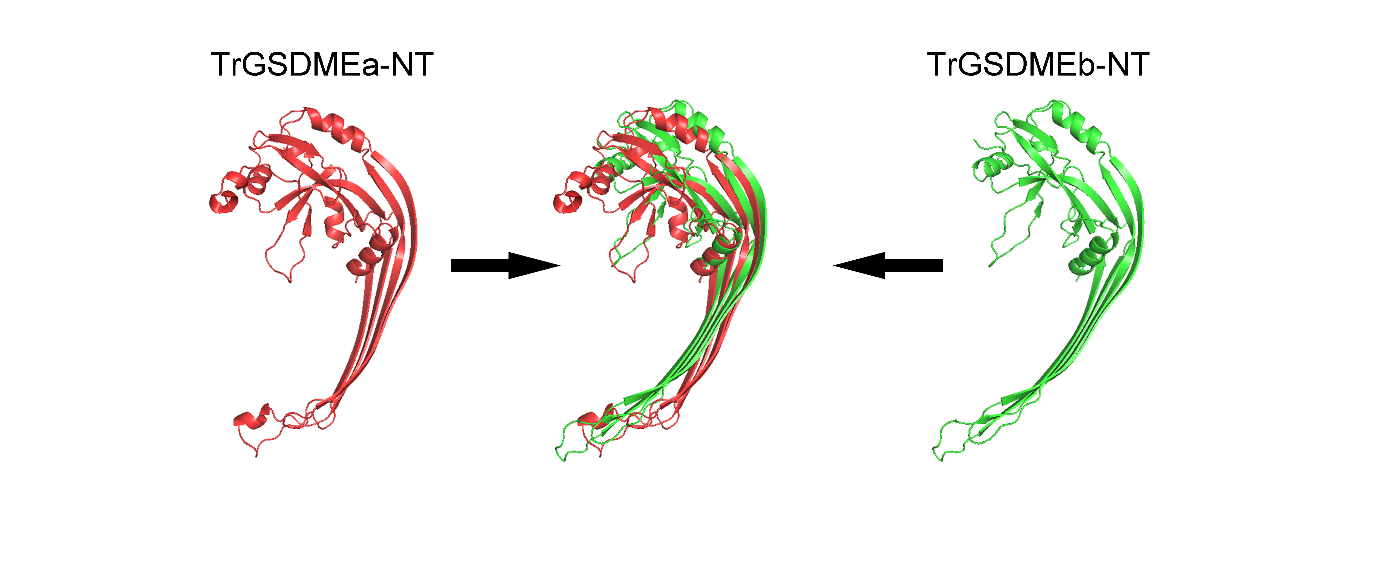
**

**Supplementary Fig. S5. The expression of TrGSDME in pufferfish tissues.** TrGSDMEa and TrGSDMEb expressions in seven pufferfish tissues were determined by qRT-PCR. Values are the means ± SD. n=3. For both TrGSDMEa and TrGSDMEb, the expression levels in intestine were set as 1.


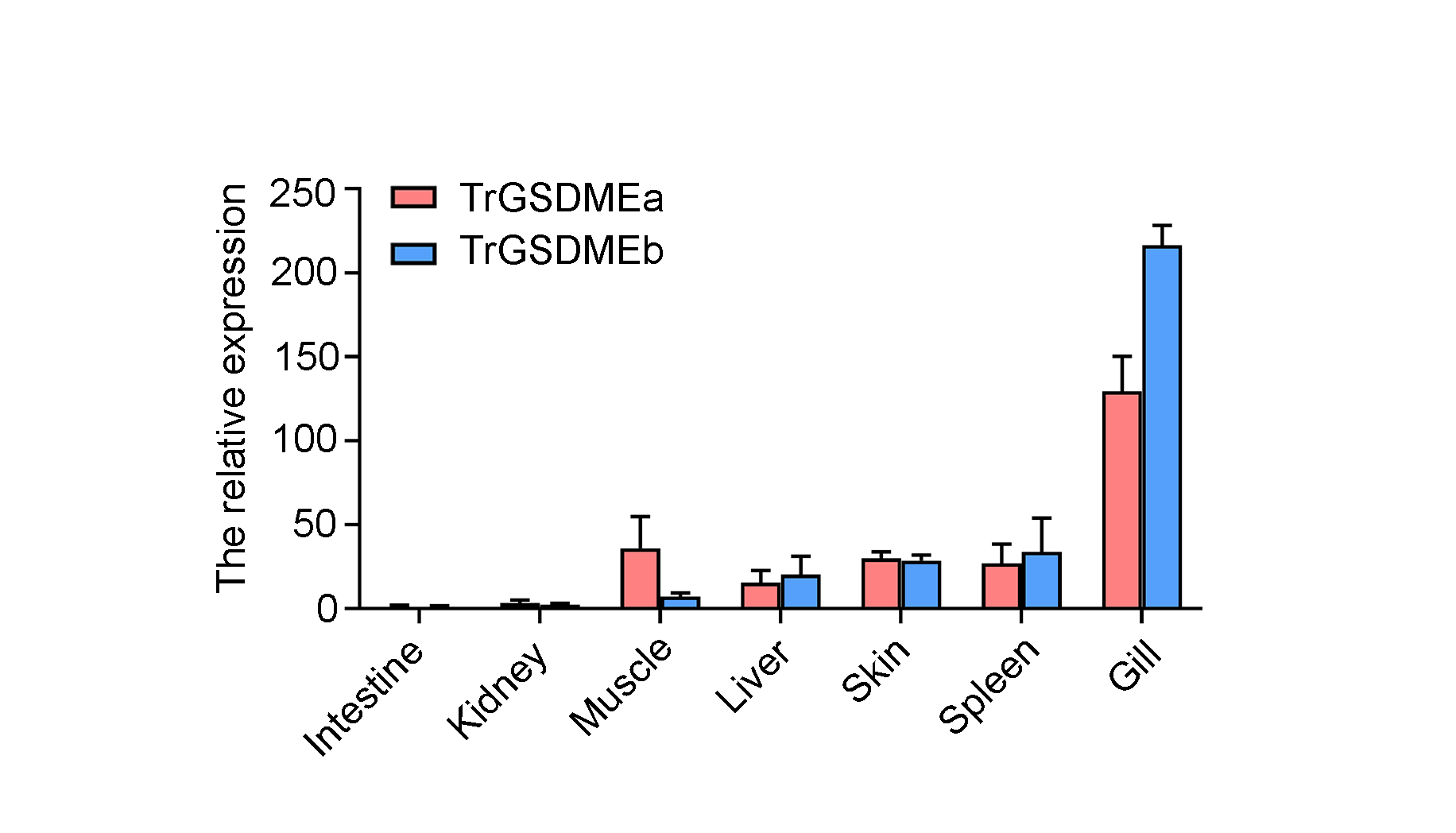


**Supplementary Fig. S6. Sequence analysis of teleost GSDMEa.** Zebrafish, turbot, and pufferfish GSDMEa (DrGSDMEa, SmGSDMEa, and TrGSDMEa, respectively) are aligned with mouse and human GSDME (MmGSDME and HsGSDME, respectively). The secondary structure is indicated on the top of the aligned sequences. The conserved amino acid residues are boxed, and the red highlights indicate 100% identity. The residues in GSDMEa-NT and GSDMEa-CT subjected to mutagenesis analysis in this study are indicated with blue and red arrows, respectively.

**
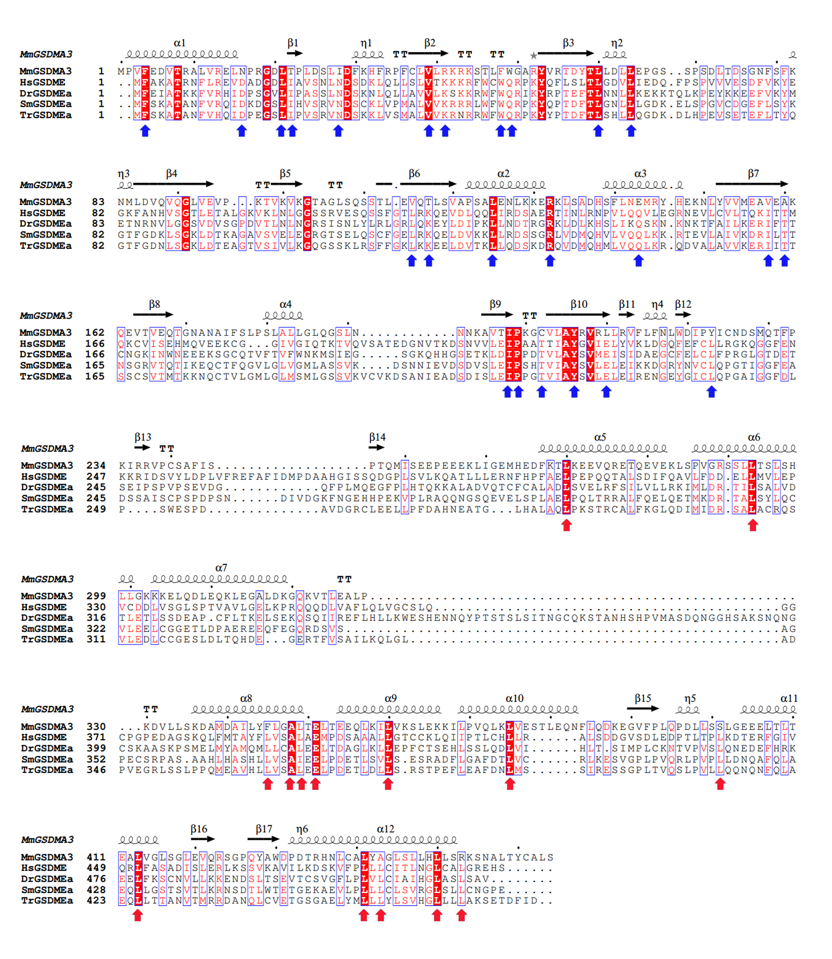
**
